# Viral metagenomics reveals the presence of novel Zika virus variants in *Aedes* mosquitoes from Barbados

**DOI:** 10.1186/s13071-021-04840-0

**Published:** 2021-06-29

**Authors:** J. Thannesberger, N. Rascovan, A. Eisenmann, I. Klymiuk, C. Zittra, H. P. Fuehrer, T. Scantlebury-Manning, M. Gittens-St.Hilaire, S. Austin, R. C. Landis, C. Steininger

**Affiliations:** 1 Division of Infectious Diseases, Department of Medicine 1, Medical University of Vienna, Spitalgasse 23, 1090 Vienna, Austria; 2 Department of Genomes & Genetics, Institut Pasteur, Paris, France; 3 Department of Cell Biology, Histology and Embryology, Gottfried Schatz Research Center, Medical University of Graz, Graz, Austria; 4 Institute of Parasitology, University of Veterinary Medicine, Vienna, Austria; 5 Department of Biological and Chemical Sciences, The University of the West Indies, Cave Hill Campus, Bridgetown, Barbados; 6 Leptospirosis Laboratory, Ministry of Health, Bridgetown, Barbados; 7 Department of Biological and Chemical Sciences, University of the West Indies, Cave Hill Campus, Cave Hill, Barbados; 8 Edmund Cohen Laboratory for Vascular Research, George Alleyne Chronic Disease Research Centre, The University of the West Indies, Bridgetown, Barbados

**Keywords:** Zika virus, Barbados, Virome, *Aedes aegypti*, Metagenomic, Arboviruses, Flaviviruses, Vector-borne diseases

## Abstract

**Background:**

The Zika virus (ZIKV) epidemic of 2015/2016 spread throughout numerous countries. It emerged in mainland Latin America and spread to neighboring islands, including the Caribbean island of Barbados. Recent studies have indicated that the virus must have already been circulating in local mosquito populations in Brazil for almost 2 years before it was identified by the World Health Organization in 2015. Metagenomic detection assays have the potential to detect emerging pathogens without prior knowledge of their genomic nucleic acid sequence. Yet their applicability as vector surveillance tools has been widely limited by the complexity of DNA populations from field-collected mosquito preparations. The aim of this study was to investigate local vector biology and characterize metagenomic arbovirus diversity in *Aedes* mosquitoes during the ongoing 2015/2016 ZIKV epidemic.

**Methods:**

We performed a short-term vector screening study on the island of Barbados during the ongoing 2015/2016 ZIKV epidemic, where we sampled local *Aedes* mosquitoes. We reanalyzed mosquito viral microbiome data derived from standard Illumina MiSeq sequencing to detect arbovirus sequences. Additionally, we employed deep sequencing techniques (Illumina HiSeq) and designed a novel bait capture enrichment assay to increase sequencing efficiency for arbovirus sequences from complex DNA samples.

**Results:**

We found that *Aedes aegypti* seemed to be the most likely vector of ZIKV, although it prevailed at a low density during the observed time period. The number of detected viruses increased with sequencing depth. Arbovirus sequence enrichment of metagenomic DNA preparations allowed the detection of arbovirus sequences of two different ZIKV genotypes, including a novel one. To our knowledge, this is the first report of the S3116W mutation in the* NS5* gene region of ZIKV polyprotein.

**Conclusions:**

The metagenomic arbovirus detection approach presented here may serve as a useful tool for the identification of epidemic-causing arboviruses with the additional benefit of enabling the collection of phylogenetic information on the source. Apart from detecting more than 88 viruses using this approach, we also found evidence of novel ZIKV variants circulating in the local mosquito population during the observed time period.

**Graphical abstract:**

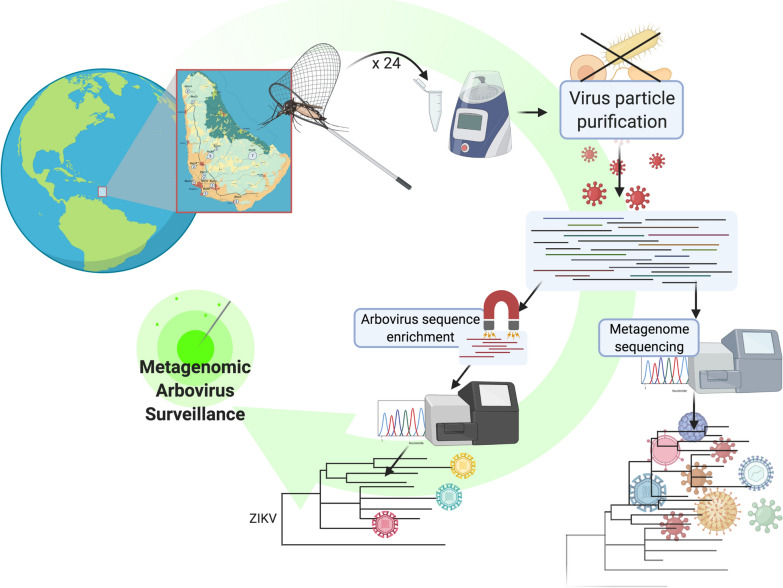

**Supplementary Information:**

The online version contains supplementary material available at 10.1186/s13071-021-04840-0.

## Background

Retrospective analysis of the Zika virus (ZIKV) epidemic of 2015/2016 showed the introduction of the Asian ZIKV lineage into Brazil from French Polynesia in 2013 [1]. The virus circulated in Brazil for almost 2 years before its identification by the World Health Organization in 2015 [2, 3]. ZIKV spread to the Caribbean in 2015, with documented ZIKV cases identified in 31 Caribbean countries by the end of 2016 [4, 5]. By the end of 2016, the number of laboratory-confirmed ZIKV cases on Barbados had risen to 147, and the number of suspected cases to 926 [6]. At the same time, the virus spread further throughout Latin America and eventually reached US governed territories (Puerto Rico, Hawaii) [4]. By the end of March 2017, ZIKV had affected at least 84 countries with more than 750,000 laboratory-confirmed ZIKV cases [3].

The relatively long delay in recognizing a clinical outbreak of a novel arbovirus emphasizes the difficulty in identifying vector-borne disease outbreaks despite existing global awareness and readiness measures. In the case of the 2015/2016 ZIKV epidemic, it took almost 4 months from the first reports of suspected arboviral infections to conclusive identification of ZIKV by the national reference laboratory [4]. The majority of known arboviruses prevail in rather narrow, circumscribed geographic regions [3]. Nevertheless, several viruses like West Nile virus (WNV), chikungunya virus or yellow fever virus (YFV), have spread globally during the past two decades [7, 8]. One possible reason for the difficulty of early culprit identification in emerging viral pandemics is the large diversity of arboviruses circulating on a global scale. The US Centers for Disease Control and Protection (CDC) lists 538 arbovirus species in their online arbovirus catalogue (https://wwwn.cdc.gov/arbocat/, 03.2018). Clinical diagnosis is difficult as most arboviruses cause similar, unspecific symptoms and serologic tests for them suffer from cross-reactivity, which reduces their specificity. Rapid arbovirus identification therefore relies on specific detection assays such as virus culture or polymerase chain reaction (PCR). However, these methods are available only in a few specialized diagnostic laboratories in Barbados. Thus, there may have been two reasons for the late diagnosis of ZIKV in Barbados: (i) the need to test for dozens of potentially circulating arboviruses; (ii) a limited availability of molecular assays for the rapid testing of a large number of clinical and environmental samples.

Hence, versatile diagnostic assays that allow both the detection of all arboviruses and those spreading to new geographic regions are urgently required. Metagenomic detection assays have the potential to detect emerging pathogens without prior knowledge of their genomic nucleic acid sequence. First, total nucleic acids present in a sample are sequenced by next-generation sequencing (NGS), and then bioinformatic analyses are used to identify viral sequences by alignment to reference genomic databases. Metagenomic analysis of clinical samples has been successfully applied to attribute several different diseases to novel infectious pathogens [9–13]. As an added benefit, NGS data may provide additional information on genetic evolution or sequence similarity with respect to viral marker genes, as we have shown in a previous study [14]. Yet, conventional whole genome NGS methods lack sensitivity for arbovirus detection because the high genetic background noise from host cells, bacteria, fungi, and other viruses reduces arbovirus-specific signals [15, 16]. This is a common challenge in virus genome sequencing from complex DNA samples such as environmental metagenomes. As shown previously, field-collected mosquito samples are likely to contain large fractions of bacterial contaminants and highly abundant environmental virus populations [17, 18]. Deep sequencing platforms have been used to enhance the sensitivity of metagenomics arbovirus detection, but increase sequencing costs [19, 20].

In this study, we aimed to comprehensively characterize the arbovirus sequence diversity in mosquitoes collected on the Caribbean island of Barbados by using a novel, cost-effective and highly sensitive NGS-based method. Barbados provides ideal breeding conditions for synanthropic mosquito species like several *Aedes* spp. because the island experiences high amounts of rainfall, especially from June to December, and has the highest population density among all the Caribbean islands. Yet, there are neither data on mosquito species prevalence nor their distribution across the island, and information on arboviruses circulating there is very limited. For this study, we reanalyzed viral metagenome sequences from genetically specified mosquito populations collected at sites across Barbados during the peak of the ZIKV epidemic in November 2016. We present evidence for the presence of two different arboviruses genotypes in the local mosquito population, including a novel variant of ZIKV.

## Methods

### Mosquito collection and taxonomic identification

Mosquito collection on Barbados was performed during the peak of the ZIKV epidemic of 2016 in the Caribbean on 8 consecutive days in October and November (Additional file 1: Table S1). General support by the local authorities was ensured before the traps were set up. Mosquitoes were sampled by a standardized method for 24-h time periods using BG-Sentinel traps equipped with carbon dioxide as an attractant (Biogents, Regensburg, Germany). Twelve collection sites were chosen across the island, all in close proximity to human housing covering core urban, urban corridor, suburban and rural areas. The collection spots represented the densely populated southwestern coastal region of the island (7/12 spots), the sparsely populated area on the northwestern coast (2/12) and the central area of the island (3/12). Trapping locations were characterized by consulting land cover maps, satellite images and photos taken at the sites. Land cover maps were provided by Barbados Town and Country Development Planning Office (National map for growth management framework 2017).

Trapped mosquitoes were shock frozen at − 80 °C within a maximum time frame of 60 min after trap disassembly and shipped on dry ice to the Medical University of Vienna research laboratory. Female mosquitoes were identified (morphologically) using the key of Becker et al. [21]. For species verification of *Aedes aegypti*, the primary vector of ZIKV, and its secondary vector, *Aedes albopictus*, genetic barcoding was performed. All of the collected *Aedes* mosquitoes (*n* = 27) were genetically analyzed by genetic barcoding the mitochondrial *COI* gene, as reported previously [22].

### Mosquito sample preparation and virus particle purification

All collected *Ae. aegypti* were pooled (*n* = 27). Five 2.8-mm ceramic beads (Peqlab, Erlangen, Germany) and 1 ml of Dulbecco’s phosphate-buffered saline (DPBS) were added prior to mechanical homogenization using a TissueLyser II (Qiagen, Venlo, the Netherlands). A virus purification and enrichment protocol (VIPEP) was performed on mosquito homogenates as described previously [14]. In brief, mosquito homogenizates were resuspended in DPBS pH 7 (no calcium, no magnesium; Thermo Fisher Scientific, Waltham, MA) to stabilize virus-like particles. Samples were then cleared of cells and cell debris by two centrifugation steps (5 min at 2500 *g* and 15 min at 4800 *g*) and subsequent filtration through a 0.45-µM syringe filter. Sample volumes of virus particle suspensions were reduced by ultrafiltration using 50-kDa molecular weight cut-off filtration units (Amicon Ultra-15 50 K; Merck Millipore, Cork, Ireland) before DNase and RNase treatment to eliminate contaminating nucleic acids. Viral DNA and RNA were isolated using a QIAamp UCP Micro Kit (Qiagen). Then, ribosomal RNA sequences were blocked and viral RNA was used for complementary DNA (cDNA) synthesis. Finally, viral genomic DNA and cDNA were concentrated by SpeedVac (Thermo Fisher Scientific) and amplified using a multiple displacement amplification-based Repli-g kit (Qiagen).

### Arboviral sequence enrichment, library preparation and NGS

We designed a custom SureSelect (Agilent Technologies, Santa Clara, CA) enrichment system targeting known arboviruses listed in the online arbovirus catalogue by CDC (https://wwwn.cdc.gov/arbocat/). As of January 2018, CDC listed 537 arbovirus species in total. Of these, 416 corresponding genomes could be identified and collected from the National Center for Biotechnology Information (NCBI) database (https://www.ncbi.nlm.nih.gov/nuccore/); 121 arboviruses had to be excluded as either no genomic sequence was found or they resembled double or wrongly annotated viruses. Arboviruses used for bait library design are listed in Additional file 2: Table S2. The bait library was designed to consist of approximately 45,200 baits with a total size of 5.8 Mb evenly covering all collected arboviral genomes. (The list of baits can be sent upon request by the author).

VIPEP-enriched genomic cDNA/DNA was quantified with a Qubit dsDNA High Sensitivity Kit on a Qubit 4 Fluorometer (Thermo Fisher Scientific) before 500 ng cDNA/DNA was fragmented by ultrasonication in a micro tube on a M220 focused ultrasonicator (Covaris, Woburn, MA). SureSelectXT HS Reagent Kit (Agilent Technologies) was used following the manufacturer’s protocols for library preparation, hybridization and capture. For quality control, sequencing libraries were quantified with the Agilent 2100 Bioanalyzer DNA High Sensitivity chip (Agilent Technologies) prior to and post-enrichment. Following target enrichment, library quantification and dilution, equimolar pools were sequenced on an Illumina MiSeq Desktop Sequencer (Illumina, San Diego, CA). Libraries were diluted to a final concentration of 8 pM and run on an entire MiSeq Flow Cell (Illumina) with version 3 chemistry in paired end mode, two times 300 bp (600 cycles chemistry) without PhiX control, according to the manufacturer’s instructions.

Shotgun library preparation on VIPEP-enriched genomic cDNA/DNA without target enrichment was done with NEBNext Ultra II DNA Library Prep Kit for Illumina [New England Biolabs (NEB) #E7645S)] in combination with the Index Primers Set 1 (NEB #7335S; New England BioLabs, Frankfurt, Germany) according to the manufacturer’s instructions. Following library preparation, equimolar pools were sequenced on Illumina MiSeq desktop and HiSeq sequencer (Illumina). For MiSeq sequencing, the same settings as above were used. For HiSeq sequencing, one lane on a HiSeq 2500 with two times 125 bp in paired end mode was used. A negative control sample (PBS buffer) was included throughout target enrichment, library prep and NGS sequencing. Validation of target enrichment was done using an artificial sample (PBS buffer), spiked with 10 ng of genomic DNA of human cytomegalovirus (HCVM), cDNA of ZIKV, influenza A virus (InfluA), WNV and YFV.

The metagenomic raw sequencing data from the MiSeq, HiSeq and target enrichment sequencing have been uploaded to the NCBI Sequence Read Archive (SRA) and can be accessed via the NCBI BioProject ID PRJNA729181. The following SRA accession numbers have been assigned: SRR14506532—MiSeq metagenomic sequences; SRR14535601—HiSeq metagenomic sequences; SRR14535600—target enrichment metagenomic sequences.

### Data assembly and sequence analysis

Raw sequencing data were quality trimmed and adaptors removed using AdapterRemoval v2.2.0, keeping only sequences longer that 30 bp, with a maximum error rate of 3 and trimming of ambiguous bases in 3′ and 5′ ends. Trimmed datasets were de novo assembled using metaSPAdes v3.12.0 with default parameters. Illumina Hi-Seq datasets were too large to be assembled with metaSPAdes, so they were assembled using megahit v1.1.1. In both cases, only contigs larger than 500 bp were used for downstream analyses. Basic Local Alignment Search Tool (BLAST) N analysis (BLAST+ v2.5.0) against the NCBI nt database, with a minimum e-value threshold of 1E−05, and retention of the first 25 hits, was used to determine contig identity. Open reading frames (ORF) were predicted on the contig sequences using MetaGeneMark v3.38. A BLASTP analysis of the predicted ORF sequences (in amino acids) against the NCBI nr database (*e*-value < 1E−05, 25 first hits) was used as a second approach. Depending on the case (see “Results” section), taxonomic annotation of contigs was resolved by either importing BLASTP results to MEGAN v6.10.13, which uses a lowest common ancestor algorithm for classification, or by considering the best BLASTP hit. The relative abundance of each contig in each sample was calculated using the reads per kilobase per million mapped reads (RPKM) metric, which normalizes data according to the contig lengths and the sequencing depth of samples. For this, reads were re-mapped on contigs using the Burrows-Wheeler aligner (BWA) v0.7.10-r789 with the aln method, and coverage and depth were calculated in the resulting BAM files using custom scripts and bedtools v2.26.0. In addition, the abundance of each individual taxa [based on the NCBI taxonomic identifier (taxid)] was also estimated using the RPKM method by using the average of RPKM of all contigs assigned to the same taxid. Reads that were not used for contig assembly were further analyzed using KrakenUniq metagenomic sequence classifier [23]. The results were collapsed at the lowest taxonomic level.

### Phylogenetic analysis

Reads matching ZIKV baits were used to query the NCBI nucleotide database using the BLASTN tool. Read pair 17,018 (reads 17,018.2RC and 17,018.1) did not have a full length 100% identity match and was used to generate the consensus sequence. To account for differences between the two paired-end sequences, only nucleotide positions that were supported by both reads were accepted for the consensus sequence. At positions that differed between the two reads, the more likely variant (i.e. identical to nucleotides at this position in most of the other ZIKV sequences) was favored. Consensus sequences of both read pairs were deposited at NCBI GenBank [accession numbers MZ126475 (17,018) and MZ126475 (21,190)].

Aligning nucleotide sequences of 11 ZIKV isolates were downloaded and used for phylogenetic tree construction. Additionally, a homologous sequence of dengue virus 2 was included as an outlier. All positions with less than 95% site coverage were eliminated. That is, fewer than 5% alignment gaps, missing data, and ambiguous bases were allowed at any position. There were a total of 97 positions in the final dataset. Phylogenetic reconstruction was performed using the maximum likelihood algorithm and Jukes-Cantor model, which was identified as the best-fitting model with the lowest Bayesian information criterion in MEGA7 [24, 25]. Initial trees for the heuristic search were obtained automatically by applying neighbor joining and BioNJ algorithms to a matrix of pairwise distances estimated using the maximum composite likelihood approach, and then selecting the topology with the superior log likelihood value. All phylogenetic analyses were conducted in MEGA7 [25].

### Virus specific real-time PCR

Real-time PCR (RT–PCR) assays were performed for the quantification of HCVM, ZIKV, InfluA, WNV and YFV as previously described [14, 26–28]. Briefly, all reactions were done in a total volume of 20 µl that contained 10 µl iTaq Universal Supermix (Bio-Rad, Hercules, CA), 300 nM of forward and reverse primers, 200 nM TaqMan probe, and 2 µl of template DNA. Mixtures were prepared in 96-well optical microtiter plates (Thermo Fisher Scientific) and amplified on a StepOnePlus Real Time PCR System (Thermo Fisher Scientific) by using the following cycling parameters: denaturation for 90 s at 95 °C, followed by 40 cycles of denaturation for 15 s at 95 °C, and annealing and extension for 60 s at 60 °C or at 57 °C (HCMV). Standards for viral quantification of HCMV and InfluA were produced as described previously [14]. For quantification of ZIKV, a 1088-bp fragment of ZIKV* NS5* gene (GI KJ776791.2; 9014–10,102) was cloned into expression vector pcDNA 3.1. (Thermo Fisher Scientific). For calculation of a standard curve and quantification of the target, we included serial dilution of plasmids within the range of 10 to 1 × 10^5^ copies/well for each RT–PCR assay. All samples were analyzed in triplicate and multiple negative controls were included in each assay.

## Results

**Fig. 1:**
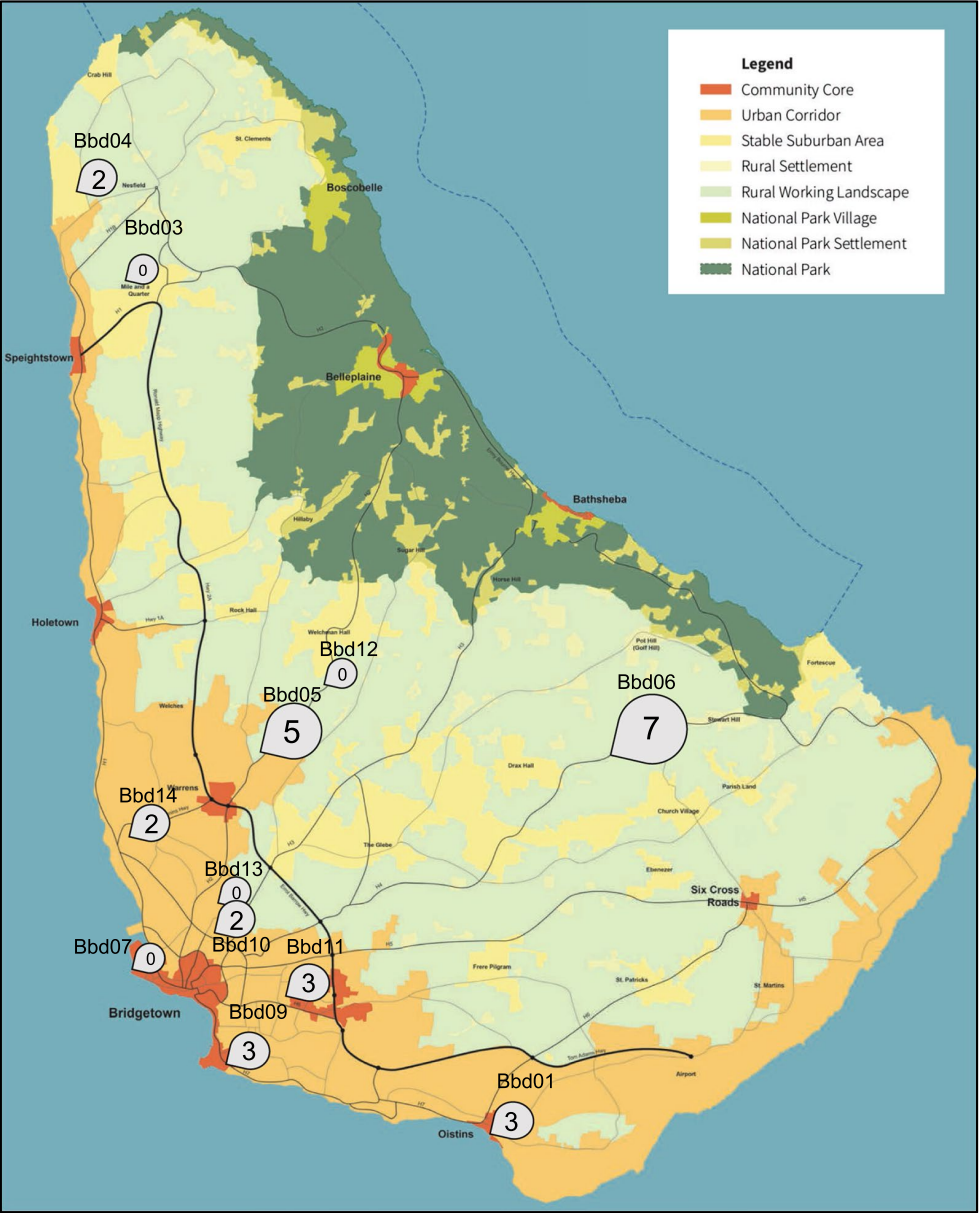
Mosquito collection spots mapped on a land cover map of Barbados.* Bubbles* indicate mosquito collection spots and number of *Aedes aegypti* individuals trapped over a period of 24 h (Barbados Town and Country Development Planning Office 2017)

We collected more than 3000 mosquitoes from October to November 2016 during the ZIKV epidemic. Among these, we identified 27 *Aedes* individuals, while the vast majority were members of the *Culex pipiens complex* (Additional file 1: Table S1). Collection spots yielding *Aedes* mosquitoes (8/12) were dispersed across the island and located in both rural and urban areas. (Fig. 1; Additional file 9: Fig. S1). All of the collected *Aedes* mosquitoes (*n* = 27) were identified as *Ae. aegypti* by mitochondrial* COI* barcoding.

Viral microbiome sequencing was performed on pooled *Ae. aegypti* mosquitoes using a previously established method to isolate intact virus particles prior to Illumina MiSeq sequencing. As a primary approach, viral metagenomic sequences were assigned by BLASTN search. Results and detailed characterization of the mosquito viral microbiome have been presented previously [18]. We did not identify any human pathogenic arboviruses among the total number of 33 viruses. To increase sequencing depth, we re-sequenced the virus-enriched *Ae. aegypti* homogenate on the Illumina HiSeq platform. Thereby we increased the total amount of raw sequencing reads to 2.9 × 10^8^. Standard BLASTN sequence assignment revealed that 90% of reads were of viral origin (Additional file 9: Fig. S2A). With increased sequencing depth, the number of detectable viruses increased to 39 (Additional file 9: Fig. S2B). We did not detect any arbovirus sequences among them.

**Fig. 2 A, B:**
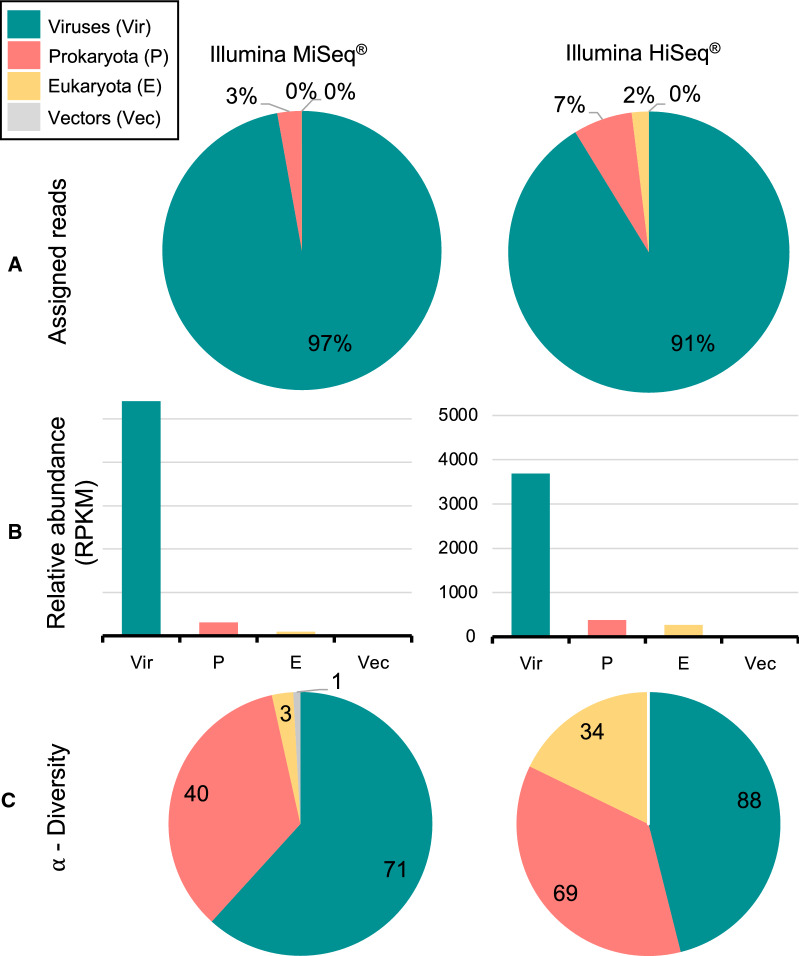
Metagenomic sequencing results after Illumina MiSeq and Illumina HiSeq sequencing. **A** Fraction of assigned reads mapped to the indicated clade by Basic Local Alignment Search Tool (BLAST) P analysis. **B** Relative abundance of reads mapped to the indicated clade, normalized to contig length and total number of reads mapped (reads per kilobase per million mapped reads;* RPKM*). **C** Richness in number of assigned clades

To enable the detection of more divergent virus sequences, metagenomic sequencing data were reanalyzed with respect to encoded protein sequences. First, metagenomic nucleotide sequences were screened for encoded ORFs. Predicted amino acid sequences were then assigned by BLASTP search. The total virus diversity detected by BLASTP analysis is presented in Additional file 3: Table S3 (MiSeq) and Additional file 4: Table S4 (HiSeq), which list the closest viral hit, phylogenetic family, associated host species and alignment quality parameters. In total, up to 97% of all protein sequences matched viral hits, and there was a high abundance of virus-derived sequences in both metagenomic datasets (Fig. 2A, B). During the analysis of protein sequences, the total number of viral hits increased from 34 to 71 in the MiSeq metagenome and from 39 to 88 in the HiSeq metagenome (Fig. 2C). However, we did not detect any hits to arboviruses.

Unassembled reads (less than 500 nt) from both sequencing runs were further classified using an algorithm for short sequence annotation (KrakenUniq metagenomic sequence classifier) [23]. We detected 5.68 × 10^6^ reads of putative viral origin (2.22 × 10^4^ for the MiSeq metagenome and 5.66 × 10^6^ for the HiSeq metagenome); however, we did not find any hits to known arbovirus species (Additional file 5: Table S5; Additional file 6: Table S6). Taxonomic results widely resembled taxonomic diversity revealed by BLASTP contig analysis.

We then enriched sequencing libraries specifically for arboviral sequences using a custom designed target enrichment system for specific capture of arbovirus sequences, and re-sequencing on the Illumina MiSeq platform. The target enrichment system has been validated by using artificially virus spiked buffer samples. The concentration of ZIKV, WNV and YFV genomic cDNA increased while non-arboviral sequences of HCMV and InfluA were eliminated (Additional file 1: Fig. S3).

**Fig. 3:**
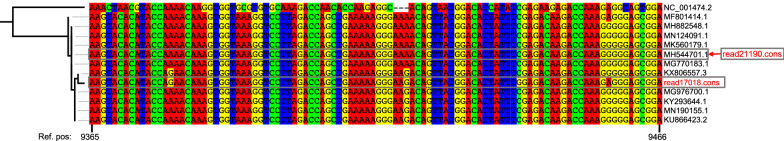
Phylogenetic relationship of novel Zika virus (ZIKV) sequences. Alignments and phylogenetic maximum likelihood tree based on the Jukes–Cantor model of 14 nucleotide sequences showing the relationship between metagenomic reads matching ZIKV bait sequences and ZIKV isolates; consensus sequence of read pair 21,190 (read21190.cons) resembles MH544701.1 sequence with 100% sequence identity; the consensus sequence of read pair 17,018 (read17018.cons; MZ126475) represents a novel ZIKV sequence, most closely related to KX806557.3; the tree was rooted to the homologous sequence of dengue virus 2, which was used as an outlier

Of the resulting 2,380,684 quality trimmed reads, only 12 reads matched bait sequences (Additional file 2: Table S2). Seven reads matched baits of sandfly fever Naples virus, one read matched baits of sandfly Sicilian turkey virus and four reads matched ZIKV bait sequences. Only the alignments of reads that matched ZIKV baits resulted in* e*-values of less than 10^–10^. The identity of bait-mapping reads was validated by using read sequences for BLAST search against the NCBI nt database. Eight reads were identified as non-viral reads of microbial origin that presumably represented contaminations, while four reads (two read pairs) could be confirmed as arboviruses (Additional file 7: Table S7). These two pairs matched ZIKV bait 0118_1x_9037 and showed the best BLASTN hit to ZIKV. Both read pairs mapped ZIKV reference genome (NC_035889) at two separate regions in the polyprotein coding sequence (9365–9466 and 9596–9701) with pairwise sequence identity between 78 and 95% (Additional file 8: Table S8). One read pair (21,190; accession number MZ126475) resembled a conserved region found in several previously described ZIKV strains, e.g. MH544701.1, MT483911.1 or MT507050.1 from Colombia and Mexico (100% pairwise sequence identity). The other two reads (read pair 17,018) did not match any NCBI sequence with 100% identity. To interrogate the taxonomic origin, we generated the consensus sequence of both reads. ZIKV isolate KX806557, which had previously been isolated in Australia, was identified as the most similar database sequence (Fig. 3). Consensus sequence 17018 (accession number MZ126475) differed from KX806557 by two nucleotide mismatches at position 9457 and 9441, resulting in amino acid point mutation S3116W of a predicted polyprotein sequence. The S3116W mutation is located within the ZIKV* NS5* gene region.

The results were validated by specific RT–PCR targeting a conserved region in the ZIKV* NS5* gene (Additional file 1: Fig. S4). The negative control sample, included throughout the target enrichment, library preparation and NGS did not yield any reads matching bait sequences. We ensured that ZIKV detection in the unenriched metagenomic datasets was not impeded by bioinformatic processing. Therefore we performed a reference-based assembly of all sequencing reads from both sequencing runs against the ZIKV reference genome (NC_035889), which resulted in zero hits.

## Discussion

The early and rapid spread of the ZIKV outbreak in 2015 to the Caribbean demonstrated that even geographically isolated islands like Barbados are vulnerable to mosquito-borne viral epidemics. Mosquito surveillance data have been collected for some islands in the Caribbean, yet limited resources and the geographic distance between islands have prohibited a comprehensive assessment of the extent of the ZIKV or any other arboviral epidemic in the Caribbean so far. We studied the mosquito virome on Barbados to assess arboviruses circulating in the environment during the local ZIKV outbreak, and identified two different ZIKV genotypes co-circulating in the local mosquito population. Viral diversity in mosquitoes may include not only different arboviruses and mosquito-specific viruses but also different strains of the same arbovirus, including novel ones.

The large number of *Cx. pipiens* complex mosquitoes collected in the present study contrasts with the low absolute and relative numbers of *Aedes* mosquitoes collected. Mosquito collection was performed when mosquito breeding was facilitated by the local rainy season during which rainfall is high (maximum 1800 mm/month) and temperatures range between 20 and 30 °C. Possible reasons for the low density of vectors are mosquito control measures and awareness programs instituted by the Barbados government, as well as changes in short-term weather conditions or variations in population dynamics. Moreover, we identified only *Ae. aegypti*, not *Ae. albopictus*, on Barbados. *Ae. aegypti* was imported into the Caribbean several centuries ago and is the primary vector for ZIKV [29], whereas *Ae. albopictus* emerged only recently on neighboring Caribbean islands and seems to be an inefficient vector for ZIKV [30]. To assess the true density and specificity of mosquito vectors, long-term vector surveillance studies are needed, which should account for temporal variations.

Metagenomic DNA sequencing is a powerful tool for non-targeted viral detection as it can be used to screen for DNA (or cDNA) sequences of viral origin without prior knowledge of sequence composition. The small *Aedes* mosquito pool available and the low relative proportion of viral sequences expected in relation to host genomes reduced the likelihood of detecting viral signals in a metagenomic study. In a previous study, we could show that enrichment of intact virus particles revealed a rich and diverse viral metagenome in *Ae. aegypti* mosquitoes [18]. However, we did not detect any arbovirus sequences. By including a MDA step in sample preparation, amplification of circular genomes is favored over that of linear nucleotide sequences such as cDNA from RNA genomes. This might impede the metagenomic detection of RNA viruses such as flaviviruses.

To ensure that arbovirus sequences were not missed by bioinformatic processing, we reanalyzed the original MiSeq metagenomic dataset. We analyzed predicted amino acid sequences and looked at short, unassembled reads, but still did not detect any arbovirus sequences. Next, we re-sequenced the sample using a deep sequencing approach (Illumina HiSeq). We found that the total number of detected viruses increased with sequencing depth. Biological significance of these metagenomic hits can be assessed only by evaluating goodness-of-assignment fit parameters. In a previous study we could show that mosquito-specific viruses and bat-associated viruses could be assigned with a high degree of confidence in this dataset [18]. Viruses of these groups are likely to establish stable infections in their mosquito hosts. Viruses associated with host organisms other than bats and mosquitoes mostly had low assignment fit parameters, indicating a high degree of novelty compared to known virus sequences. Hence, host assignments of these hits might be misleading. It cannot be concluded from metagenomic data alone whether these viruses represent possible vector-borne ones or ones passing through the mosquito.

Even though the total number of viruses detected by Illumina HiSeq sequencing increased to 88, arbovirus sequences remained undetectable. Direct sequencing of virus-enriched nucleic acids yields representative proportions of sequence reads mapping viral target genomes. Large viral DNA and cDNA populations that include highly abundant non-target sequence entities impede the sequencing of low-abundant target genomes. Virus genome sequencing studies commonly rely on multiplex PCR to amplify minute sequence entities from complex DNA preparations. However, this method can affect sequencing results by introducing nucleotide mutations or gene deletions [31, 32]. Target sequences with mutated primer binding sites are not amplified by multiplex PCR, and often remain discriminated by NGS [15, 33, 34]. Target sequence capture by hybridization to bait RNA libraries has proven highly effective for virus genome sequencing of challenging samples [35, 36].

Thus, we designed approximately 45,200 biotinylated RNA baits to cover 416 arbovirus genomes. Hybridization of library-prepared nucleic acids to RNA baits enables the isolation and enrichment of sequences of interest from other nucleic acids. Arbovirus-specific target enrichment allowed the detection of ZIKV reads, which was not possible with Miseq and HiSeq sequencing approaches. By considering even short sequences of less than 500 nucleotides and performing a mapped assembly to the ZIKV reference genome we could rule out that possible ZIKV reads were missed by the bioinformatic data processing. Two ZIKV cDNA sequences matched previously described virus isolates while the other two reads exhibited only partial identity to any ZIKV database entry. Our results indicate the presence of a novel ZIKV sequence together with a previously described fragment in the small pool of *Ae. aegypti* mosquitoes evaluated. As both read pairs align at different regions of the ZIKV genome we cannot conclude whether the fragments originate from a mutual ZIKV strain or indicate co-circulation of at least two variants. However, the predicted amino acid point mutation S3116W encoded by the novel sequence is located within the* NS5* gene region of the ZIKV polyprotein. The* NS5* gene has been shown to play a crucial role in ZIKV immune evasion and in ZIKV gene translation [37–39]. To our knowledge, this is the first study to report the S3116W mutation. The impact of the S3116W mutation on* NS5* gene function must be further elucidated.

The remaining eight out of 12 sequences that were enriched by arbovirus target enrichment showed only short stretches of sequence similarities (down to 35 out of 120 nucleotides) to corresponding bait sequences. Bait capture target enrichment allows enrichment of low-abundance sequences, even when nucleotide sequences of metagenomic targets only partly match the bait library. Arbovirus target enrichment is a highly suitable tool with which to increase the efficiency and sensitivity of metagenomic studies in various applications. Though the processing costs of single reactions are increased by target enrichment, higher detection sensitivity allows the testing of larger pool sizes. This is of special benefit to vector surveillance programs that have to analyze large pools of arthropods to remain economically feasible. One potential drawback of this method is that bait libraries have to be updated regularly to include newly identified arbovirus species. However, we were able to show that nucleotide sequences as opposed to bait sequences with up to 70% difference can still be enriched. Thus, it can be assumed that novel genetic variants of known arboviruses remain detectable without having to adapt the bait library.

ZIKV was the only arbovirus that we detected among the numerous other arboviruses that have been documented to circulate in the Caribbean (WNV, dengue virus, chikungunya virus, etc.). We may not have detected some arboviruses because of the small mosquito pool available. Nevertheless, our results indicate that, during an arbovirus outbreak, the virus in question circulates in the environment at a higher abundance than the others. To our knowledge, this observation has not been studied in previous work, but could be taken into account for the early identification of the causal virus of an outbreak. Small numbers of mosquito individuals are sufficient for arbovirus surveillance by metagenomic virome sequencing. Identification of viruses from clinical samples is problematic because of non-specific symptoms, serological cross-reactivity between arboviruses and short periods of viremia. In contrast, metagenomic detection of arbovirus species that circulate at high abundance in a local mosquito population could facilitate more rapid identification of the causal agent of viral outbreaks, and provide additional phylogenetic information on circulating arboviruses.

## Conclusions

In conclusion, we found evidence that *Ae. aegypti* is most probably the predominant vector for ZIKV throughout the island of Barbados, although it was present at a low density during the short observation period of the study. However, we cannot rule out the local occurrence of other competent mosquito vectors of ZIKV. Metagenome sequencing of *Ae. aegypti* mosquitoes revealed that the mosquito viral microbiome was highly diverse and included viruses from many different reservoirs. A large diversity of viruses other than arboviruses prevailed at a high level within these complex cDNA/DNA populations. We reanalyzed *Ae. aegypti* metagenomic virome datasets in this study to find arbovirus-derived sequences. Use of amino acid sequence alignment and deep sequencing technology increased the total number of detected viruses but did not facilitate arbovirus sequence detection. Only by specific arbovirus capture technology were we able to identify arbovirus sequences. We gathered evidence from this relatively small pool of mosquitoes for the existence of different ZIKV genotypes, including a novel one. Metagenomic screening of mosquito populations may be a useful tool for identifying the viral culprit of arbovirus outbreaks and for the collection of phylogenetic information on the source of an outbreak.

## Supplementary Information

**Additional file 1: Table S1.** Locations of mosquito collection; 24-h period, BG Sentinel traps.

**Additional file 2: Table S2.** Virus list used for bait library design of arbovirus-specific target enrichment.

**Additional file 3: Table S3.*** Aedes aegypti* virome results (MiSeq).

**Additional file 4: Table S4.** *Aedes aegypti* virome results (HiSeq).

**Additional file 5: Table S5.** Results of KrakenUniq analysis of unassembled reads from MiSeq metagenome.

**Additional file 6: Table S6.** Results of KrakenUniq analysis of unassembled reads from HiSeq metagenome.

**Additional file 7: Table S7.** Results of arbovirus target enrichment with reads mapping bait sequences and corresponding BLASTN hit.

**Additional file 8: Table S8.** Mapping target enrichment-derived reads to Zika virus (*ZIKV*) reference genome (NC_012532.1).

**Additional file 9: Figure S1.** Local environment at mosquito trapping spots characterized by satellite imagery (Google maps); photographs taken by the authors during trap assembly. **Figure S2.** Metagenomic sequencing results from Illumina HiSeq sequencing. **a** Fraction of assigned reads mapped to the indicated clade in the Basic Local Alignment Search Tool (BLAST) N analysis; **b** relative abundance of reads mapped to indicated clade, normalized to contig length and number of reads mapped in total (reads per kilobase per million mapped reads;* RPKM*); **c** richness in number of assigned clades. **Figure S3.** **a** Absolute and **b** relative quantification by real-time polymerase chain reaction (RT–PCR) assays of artificially spiked viruses before and after target enrichment (*TE*); human cytomegalovirus (*HCMV*), ZIKV, influenza virus (*InfluA*), West Nile virus (*WNV*), yellow fever virus (*YFV*). **Figure S4.** Quantitative RT–PCR targeting a conserved region in the ZIKV* NS5* gene; ZIKV load in the unenriched library and in the arbovirus specific target-enriched library of pooled *Ae. aegypti* mosquitos (*n* = 27). Measurements were made for biological triplicates.* Error bars* indicate SEM.

## Data Availability

All metagenomic datasets will be made available on the NCBI Sequence Read Archive upon publication of this article. Bait sequences are available from the corresponding author.
