# Supplementary material for: Viral metagenomics reveals the presence of novel Zika virus variants in Aedes mosquitoes from Barbados
Source: Parasit Vectors. 2021 Jun 29;14:343. doi: 10.1186/s13071-021-04840-0 (PMC8244189; doi:10.1186/s13071-021-04840-0)
Supplement: Supplementary file 1 — Additional file 1: Table S1. Locations of mosquito collection; 24-h period, BG Sentinel traps. [file 13071_2021_4840_MOESM1_ESM.docx]

**Table S1** Locations of mosquito collection; 24h period, BG Sentinel traps

| **sample ID** | **Latitude/Longitude** | **date** | **Description** |  | **core urban** | **urban corridor** | **suburban** | **rural** |  | **Ae. aegypti** | **Ae. vexans** | **Cx.pip. compl.** |
| --- | --- | --- | --- | --- | --- | --- | --- | --- | --- | --- | --- | --- |
| Bbd01 | 13.0622196197509/ -59.5383224487304 | 31.10.16 | private garden |  | x |  |  |  |  | 3 |  | x |
| Bbd03 | 13.2693939208984/ -59.6246032714843 | 31.10.16 | private garden |  |  |  |  | x |  |  |  | x |
| Bbd04 | 13.298430442810059/ -59.64463424682617 | 02.11.16 | private garden |  |  |  | x |  |  | 2 |  |  |
| Bbd05 | 13.150904655456543/ -59.596473693847656 | 03.11.16 | private garden |  |  |  |  | x |  | 5 |  |  |
| Bbd06 | 13.155024528503418/ -59.5092887878418 | 03.11.16 | farm |  |  |  |  | x |  | 7 |  | x |
| Bbd07 | 13.1006937 02697754/ -59.6285514831543 | 04.11.16 | port |  | x |  |  |  |  |  |  | x |
| Bbd09 | 13.078596944444444/ -59.60511 | 05.11.16 | private garden |  | x |  |  |  |  | 3 |  |  |
| Bbd10 | 13.108885833333334/ -59.60619333333333 | 05.11.16 | private garden |  |  | x |  |  |  | 2 |  |  |
| Bbd11 | 13.0941524505615/ -59.5905647277832 | 06.11.16 | private garden |  |  | x |  |  |  | 3 |  |  |
| Bbd12 | 13.169263333333300/ -59.58374833333333 | 06.11.16 | private garden |  |  |  |  | x |  |  |  | x |
| Bbd13 | 13.11695388888880/ -59.60735666666667 | 07.11.16 | private garden |  |  | x |  |  |  |  |  | x |
| Bbd14 | 13.133648888888889/ -59.63046333333333 | 07.11.16 | university campus |  |  | x |  |  |  | 2 |  |  |
