# Supplementary material for: Viral metagenomics reveals the presence of novel Zika virus variants in Aedes mosquitoes from Barbados
Source: Parasit Vectors. 2021 Jun 29;14:343. doi: 10.1186/s13071-021-04840-0 (PMC8244189; doi:10.1186/s13071-021-04840-0)
Supplement: Supplementary file 7 — Additional file 7: Table S7. Results of arbovirus target enrichment with reads mapping bait sequences and corresponding BLASTN hit. [file 13071_2021_4840_MOESM7_ESM.docx]

**Table S7** Results of arbovirus target enrichment with reads mapping bait sequences and corresponding BLASTN hit

|  |  |  |  |  |  | **Best BLASTN Hit** | | | | |
| --- | --- | --- | --- | --- | --- | --- | --- | --- | --- | --- |
| **Bait target** | **Read ID** | **Alignment Length (nt)** | **% ID** | **E-value** | **Score** | **Hit** | **Alignment length** | **% ID** | **Score** | **E-value** |
| NC_012532.1 Zika virus | 17018:1 | 101 | 78.218 | 1.39E-17 | 92 | KX806557.3 Zika virus isolate TS17-2016 | 101 | 87 | 123 | 6.00E-25 |
|  | 17018:2 | 99 | 87.879 | 4.53E-30 | 138 | KX806557.3 Zika virus isolate TS17-2016 | 101 | 98 | 178 | 3.00E-41 |
|  | 21190:1 | 94 | 94.681 | 1.39E-36 | 162 | MH544701.1 Zika virus isolate 459148_2016 | 101 | 100 | 183 | 7.00E-43 |
|  | 21190:2 | 101 | 95.050 | 2.20E-40 | 176 | MH544701.1 Zika virus isolate 459148_2016 | 101 | 100 | 183 | 7.00E-43 |
| HM566170.1 Sandfly fever Naples virus |  | 35 | 88.571 | 3.49E-06 | 50 | CP022656.1 Delftia acidovorans strain RAY209 genome | 101 | 98 | 174 | 4.00E-40 |
|  |  | 35 | 88.571 | 3.49E-06 | 50 | CP022656.1 Delftia acidovorans strain RAY209 genome | 101 | 96 | 165 | 2.00E-37 |
|  |  | 35 | 88.571 | 3.49E-06 | 50 | CP022656.1 Delftia acidovorans strain RAY209 genome | 101 | 97 | 168 | 2.00E-38 |
|  |  | 35 | 88.571 | 3.49E-06 | 50 | CP022656.1 Delftia acidovorans strain RAY209 genome | 101 | 98 | 174 | 4.00E-40 |
|  |  | 35 | 88.571 | 3.49E-06 | 50 | CP022656.1 Delftia acidovorans strain RAY209 genome | 101 | 98 | 174 | 4.00E-40 |
|  |  | 35 | 88.571 | 3.49E-06 | 50 | CP022656.1 Delftia acidovorans strain RAY209 genome | 101 | 98 | 174 | 4.00E-40 |
|  |  | 35 | 88.571 | 3.27E-06 | 50 | CP022656.1 Delftia acidovorans strain RAY209 genome | 94 | 95 | 174 | 5.00E-32 |
| NC_015411.1 Sandfly Sicilian Turkey virus |  | 70 | 77.143 | 7.89E-08 | 56 | CP015055.1 Kluyveromyces marxianus strain CBS4857 chromosome 2 sequence | 77 | 87 | 95.1 | 3.00E-16 |
