# Supplementary material for: Viral metagenomics reveals the presence of novel Zika virus variants in Aedes mosquitoes from Barbados
Source: Parasit Vectors. 2021 Jun 29;14:343. doi: 10.1186/s13071-021-04840-0 (PMC8244189; doi:10.1186/s13071-021-04840-0)
Supplement: Supplementary file 8 — Additional file 8: Table S8. Mapping target enrichment-derived reads to Zika virus (ZIKV) reference genome (NC_012532.1). [file 13071_2021_4840_MOESM8_ESM.docx]

**Table S8** Mapping target enrichment derived reads to Zika Virus reference genome (NC_012532.1)

|  |  |  | **Position on NC_012532.1** | | **Alignment parameters** | | |
| --- | --- | --- | --- | --- | --- | --- | --- |
| **Read ID** | **Read length** | **% aligned** | **Start** | **End** | **% ID** | **E-value** | **score** |
| 17018:1 | 101 | 100 | 9365 | 9465 | 78 | 4,00E-20 | 84.2 |
| 17018:2 | 101 | 98 | 9368 | 9466 | 88 | 1,00E-32 | 125 |
| 21190:1 | 101 | 98 | 9596 | 9694 | 95 | 8,00E-42 | 157 |
| 21190:2 | 101 | 100 | 9601 | 9701 | 95 | 6,00E-43 | 160 |
