# Supplementary material for: Viral metagenomics reveals the presence of novel Zika virus variants in Aedes mosquitoes from Barbados
Source: Parasit Vectors. 2021 Jun 29;14:343. doi: 10.1186/s13071-021-04840-0 (PMC8244189; doi:10.1186/s13071-021-04840-0)
Supplement: Supplementary file 9 — Additional file 9: Figure S1. Local environment at mosquito trapping spots characterized by satellite imagery (Google maps); photographs taken by the authors during trap assembly. Figure S2. Metagenomic sequencing results from Illumina HiSeq sequencing. a Fraction of assigned reads mapped to the indicated clade in the Basic Local Alignment Search Tool (BLAST) N analysis; b relative abundance of reads mapped to indicated clade, normalized to contig length and number of reads mapped in total (reads per kilobase per million mapped reads; RPKM); c richness in number of assigned clades. Figure S3. a Absolute and b relative quantification by real-time polymerase chain reaction (RT–PCR) assays of artificially spiked viruses before and after target enrichment (TE); human cytomegalovirus (HCMV), ZIKV, influenza virus (InfluA), West Nile virus (WNV), yellow fever virus (YFV). Figure S4. Quantitative RT–PCR targeting a conserved region in the ZIKV NS5 gene; ZIKV load in the unenriched library and in the arbovirus specific target-enriched library of pooled Ae. aegypti mosquitos (n = 27). Measurements were made for biological triplicates. Error bars indicate SEM. [file 13071_2021_4840_MOESM9_ESM.pdf]

Figure S1

	Satellite (Google maps)	Local setting	
Bbd01	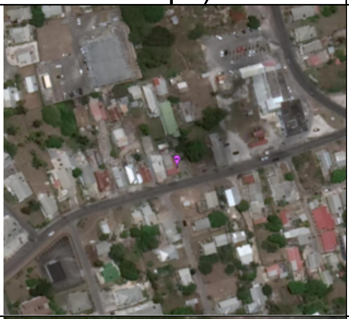	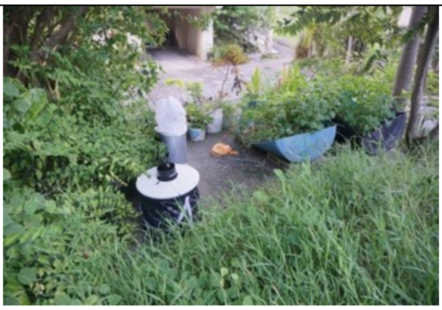	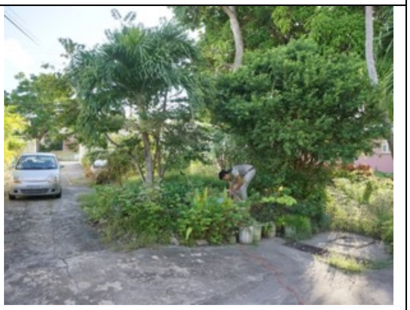
Bbd03	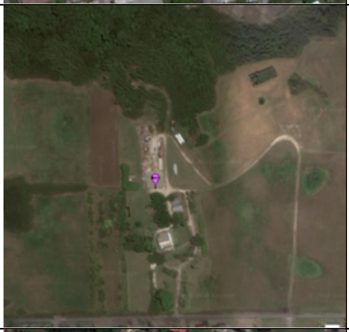	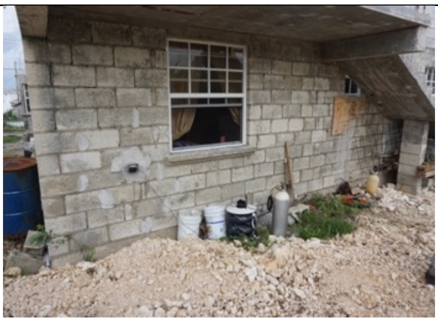	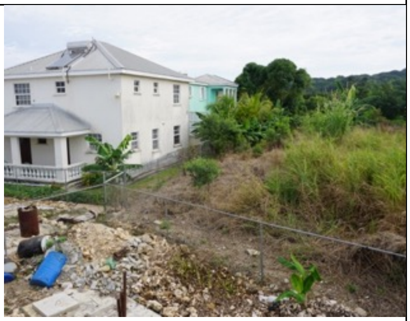
Bbd04	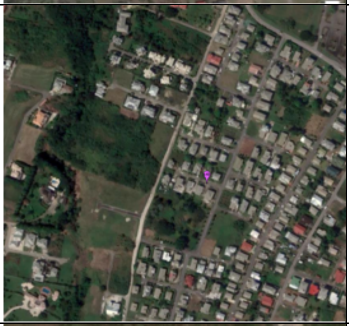	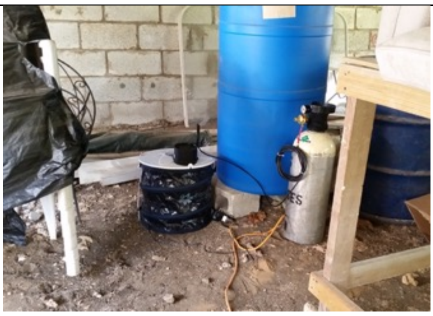	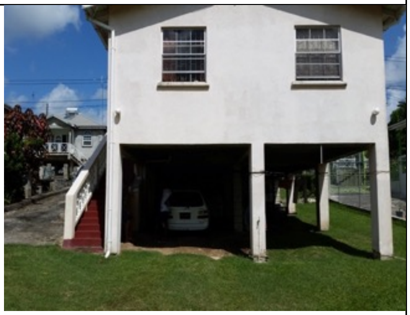
Bbd05	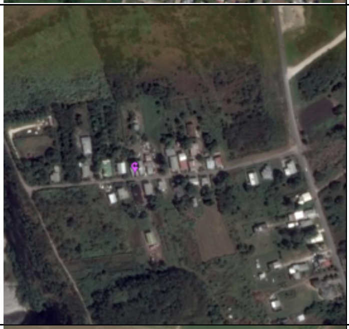	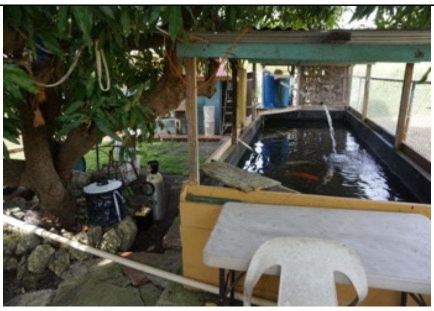	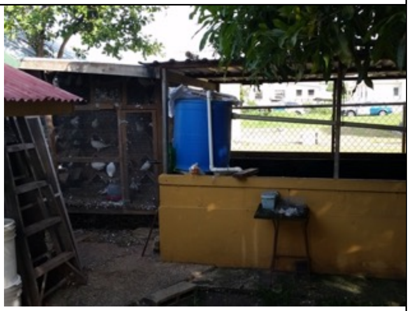
Bbd06	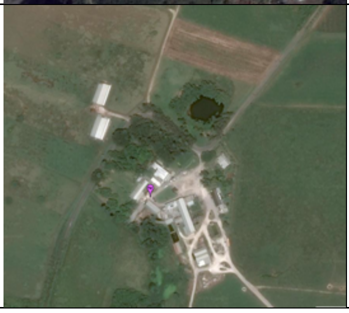	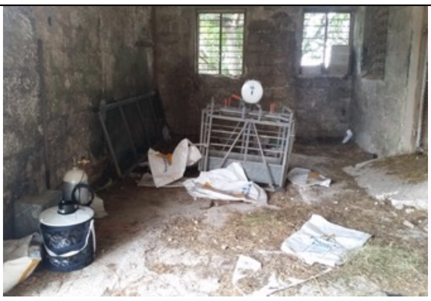	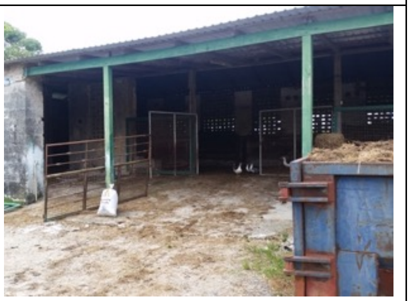

Bbd07	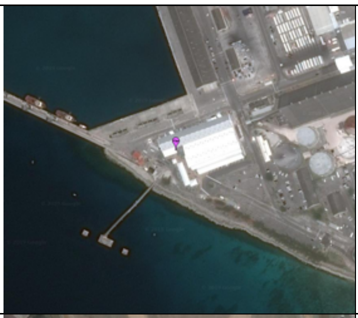	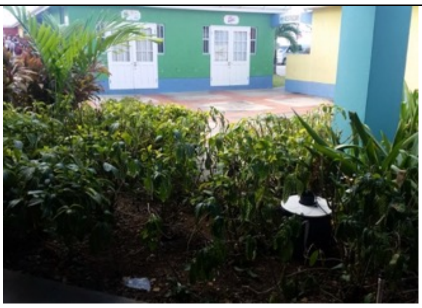	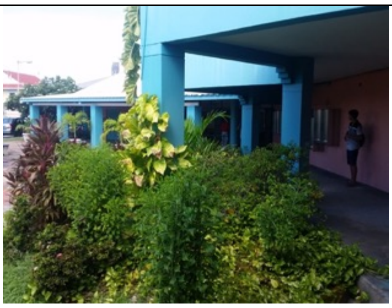
Bbd09	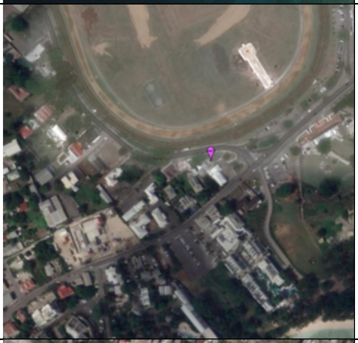	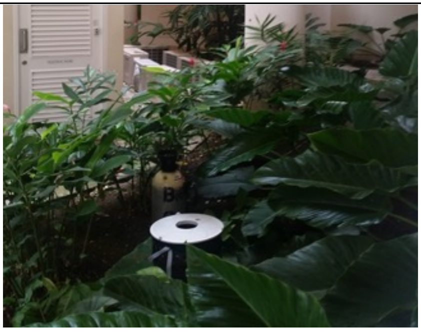	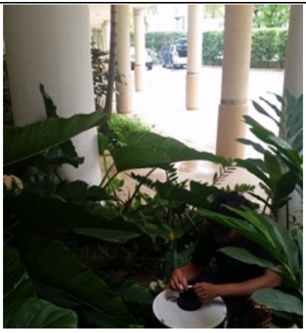
Bbd10	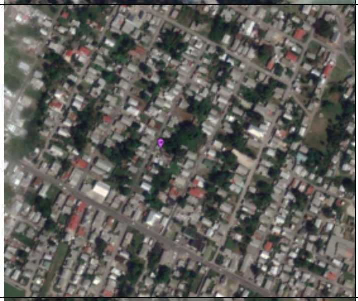	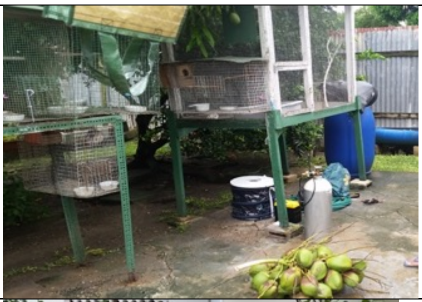	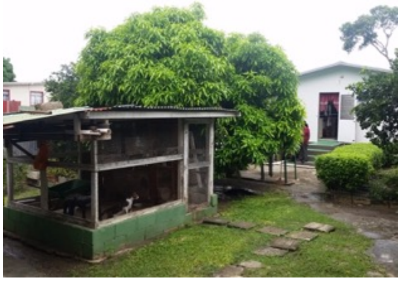
Bbd11	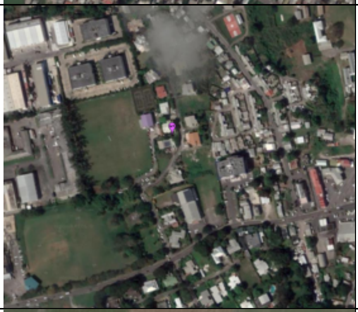	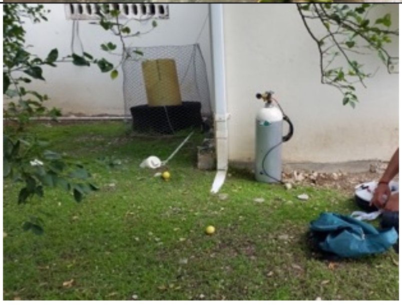	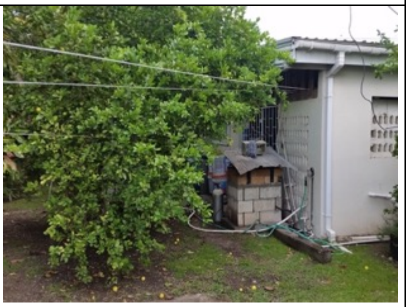
Bbd12	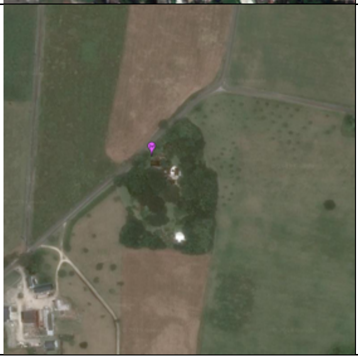	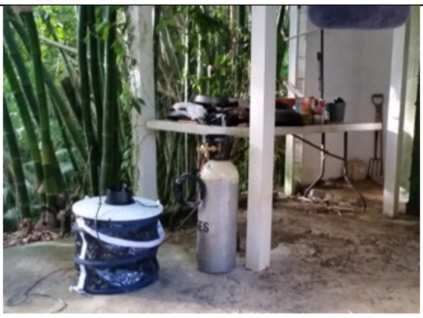	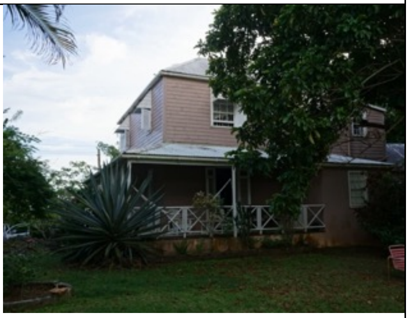

Bbd13	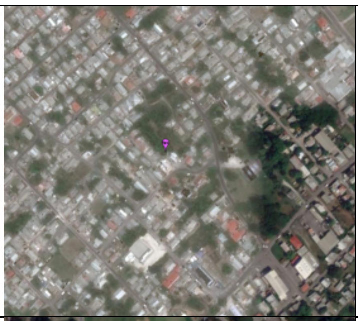	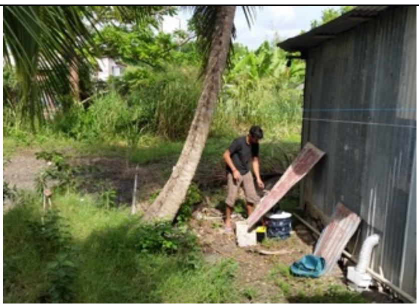	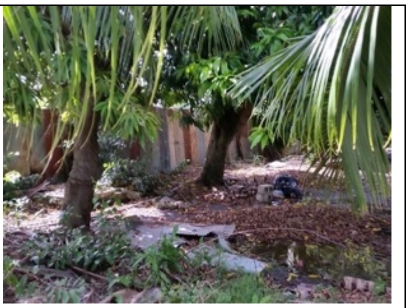
Bbd14	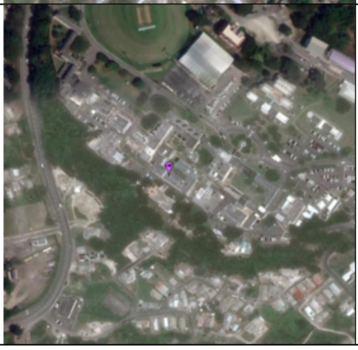	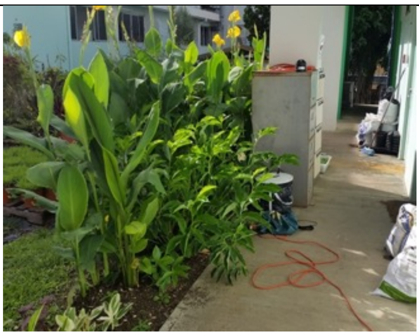	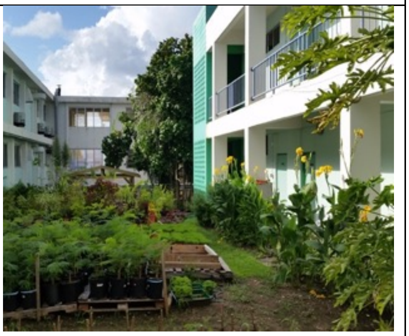

Figure S2

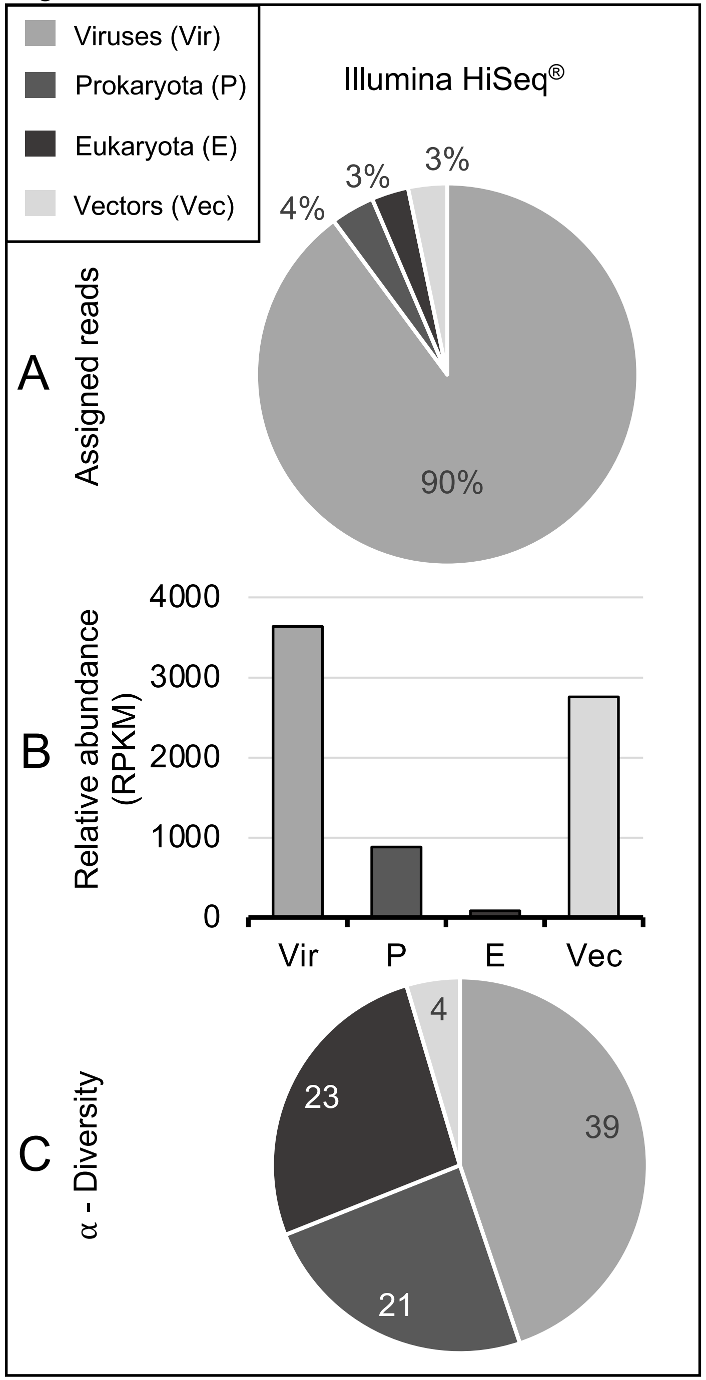

Viruses (Vir)
Prokaryota (P)
Eukaryota (E)
Vectors (Vec)
Illumina HiSeq®
A
Assigned reads
4%
3%
3%
90%
B
Relative abundance (RPKM)
4000
3000
2000
1000
0
Vir
P
E
Vec
C
α - Diversity
4
23
39
21

Figure S3

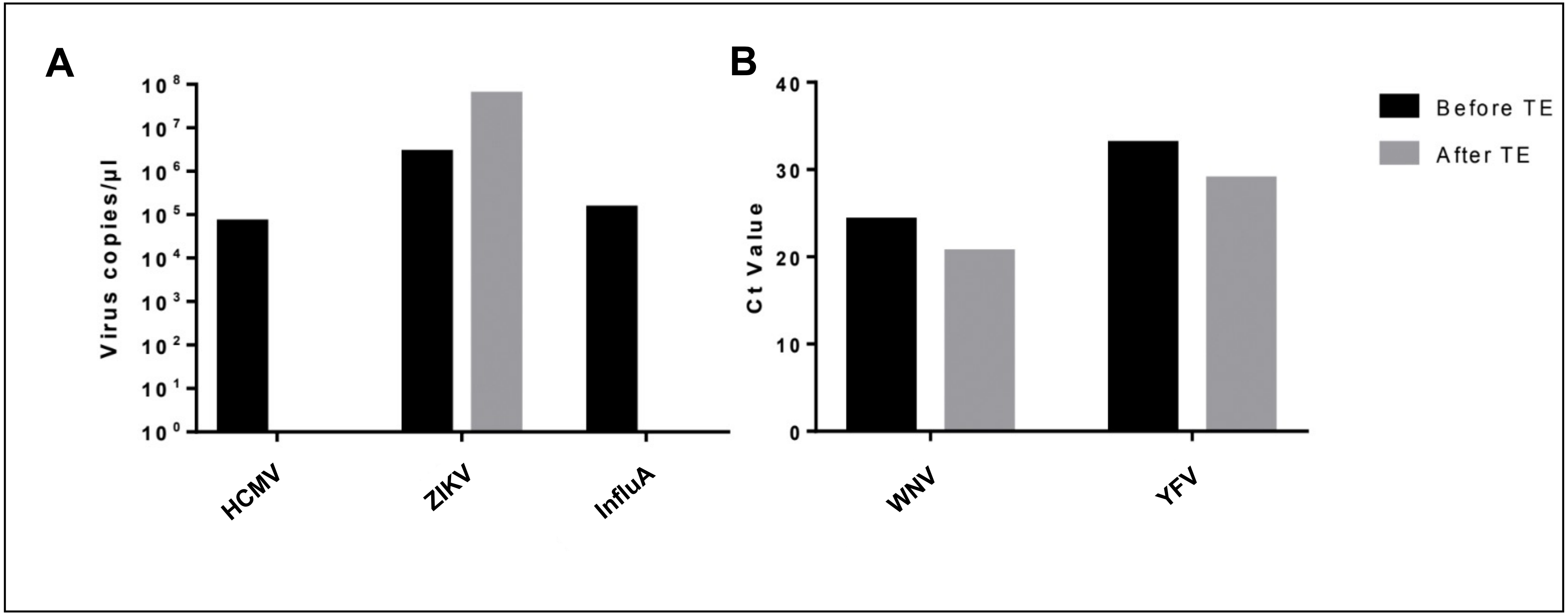
A
B
Virus copies/µl
10^8
10^7
10^6
10^5
10^4
10^3
10^2
10^1
10^0
HCMV
ZIKV
InfluA
Ct Value
40
30
20
10
0
WNV
YFV
Before TE
After TE

Figure S4

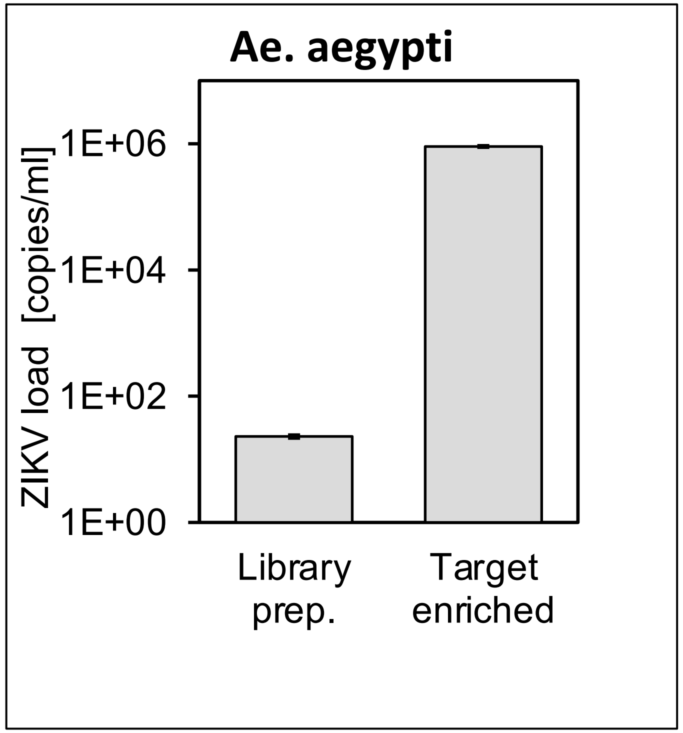
Ae. aegypti
ZIKV load [copies/ml]
1E+06
1E+04
1E+02
1E+00
Library prep.
Target enriched
